# Supplementary material for: Predation risk of the sea urchin Paracentrotus lividus juveniles in an overfished area reveal system stability mechanisms and restocking challenges
Source: PLoS One. 2024 Apr 18;19(4):e0301143. doi: 10.1371/journal.pone.0301143 (PMC11025834; doi:10.1371/journal.pone.0301143)
Supplement: S1 Table — (DOCX) [file pone.0301143.s001.docx]

**Table S1 List of algae composing turfs in experimental area.**

| **list of algae** |
| --- |
| *Acetabularia acetabulum* (Linnaeus) P.C.Silva 1952 |
| *Acrothamnion preissii* (Sonder) E.M.Wollaston 1968 |
| *Amphiroa rigida* J.V.Lamouroux 1816 |
| *Antithamnion sp.* Nägeli, 1847 |
| *Caulerpa cylindracea* Sonder 1845 |
| *Cladophora* (crf) *laetevirens* (Dillwyn) Kützing 1843 |
| *Cladophora prolifera* (Roth) Kützing 1843 |
| *Cutleria multifida* (sporofite) (Turner) Greville 1830 |
| *Cystoseira* sp. C. Agardh, 1820 |
| *Dasya rigidula* (Kützing) Ardissone 1878 |
| *Dictyota dichotoma* var. *intricata* (C.Agardh) Greville 1830 |
| *Ellissolandia elongata* (J.Ellis & Solander) K.R.Hind & G.W.Saunders, 2013 |
| *Flabellia petiolata* (Turra) Nizamuddin 1987 |
| *Halopithys incurva* (Hudson) Batters 1902 |
| *Halopteris scoparia* (Linnaeus) Sauvageau 1904 |
| *Halydictyon mirabile* Zanardini 1843 |
| *Herposiphonia secunda* (C.Agardh) Ambronn 1880 |
| *Jania virgata* (Zanardini) Montagne 1846 |
| *Laurencia obtusa* (Hudson) J.V.Lamouroux, 1813 |
| *Padina pavonica* (Linnaeus) Thivy 1960 |
| *Polysiphonia elongata* (Hudson) Sprengel, 1827 |
| *Polysiphonia scopulorum* Harvey, 1855 |
| *Sphacelaria cirrosa* (Roth) C.Agardh, 1824 |
| *Vertebrata fruticulosa* (Wulfen) Kuntze, 1891 |
| *Wrangelia penicillata* C.Agardh, 1828 |
