# Supplementary material for: Predation risk of the sea urchin Paracentrotus lividus juveniles in an overfished area reveal system stability mechanisms and restocking challenges
Source: PLoS One. 2024 Apr 18;19(4):e0301143. doi: 10.1371/journal.pone.0301143 (PMC11025834; doi:10.1371/journal.pone.0301143)
Supplement: S2 Table — The asterisk indicates the predators of Paracentrotus lividus (Sala, 1997; Guidetti 2004; Bonaviri et al., 2010). (DOCX) [file pone.0301143.s002.docx]

**Table S2 Average abundance (± SE) in 125 m^2^ of fish in the experimental area depending on their size: individuals smaller (macro-predators, that potentially enter the cages) and larger (mega-predators) than 2 cm in body length.** The asterisk indicates the predators of *Paracentrotus lividus* (Sala, 1997; Guidetti 2004; Bonaviri et al., 2010).

| **Fish species** | **Macro-predator**  **size** | **Mega-predator**  **size** |
| --- | --- | --- |
| *Chromis chromis* | 0 | 27±10.8 |
| **Coris julis* | 13±1.7 | 0 |
| **Diplodus annularis* | 0 | 4±1.5 |
| **Diplodus puntazzo* | 0 | 0±0.2 |
| **Diplodus sargus* | 0 | 2±1.7 |
| **Diplodus vulgaris* | 0 | 5±1.5 |
| **Labrus merula* | 0 | 0±0.2 |
| *Oblada melanura* | 0 | 6±3.8 |
| *Sarpa salpa* | 0 | 17±8.5 |
| *Scorpaena porcus* | 0 | 0±0.1 |
| **Serranus cabrilla* | 0 | 1±0.3 |
| **Serranus scriba* | 0 | 1±0.4 |
| **Sparus aurata* | 0 | 1±0.6 |
| *Spondyliosoma cantharus* | 0 | 1±0.3 |
| *Symphodus mediterraneus* | 0 | 0±0.2 |
| *Symphodus melanocercus* | 0 | 0±0.2 |
| *Symphodus ocellatus* | 0 | 0±0.2 |
| *Symphodus roissali* | 0 | 0±0.1 |
| *Symphodus rostratus* | 0 | 1±0.4 |
| *Symphodus tinca* | 0 | 11±2.5 |
| **Thalassoma pavo* | 0 | 0±0.3 |
