# Supplementary material for: Predation risk of the sea urchin Paracentrotus lividus juveniles in an overfished area reveal system stability mechanisms and restocking challenges
Source: PLoS One. 2024 Apr 18;19(4):e0301143. doi: 10.1371/journal.pone.0301143 (PMC11025834; doi:10.1371/journal.pone.0301143)
Supplement: S3 Table — The asterisk indicates the general carnivores. (DOCX) [file pone.0301143.s003.docx]

**Table S3 List of macrofauna species** **found in both Turf substrate and Barren substrate in the experimental area.** The asterisk indicates the general carnivores.

| **TURF** | **BARREN** |
| --- | --- |
| **Mollusca Bivalvia** | **Mollusca Bivalvia** |
| *Parvicardium exiguum* (Gmelin, 1791) | *Glans trapezia* (Linnaeus, 1767) |
| *Abra alba* (W. Wood, 1802) | *Gouldia minima* (Montagu, 1803) |
| *Barbatia barbata* (Linnaeus, 1758) |  |
|  | **Mollusca Gastropoda** |
| **Mollusca Gastropoda** | *Bittium latreillii* (Payraudeau, 1826) |
| *Alvania cimex* (Linnaeus, 1758) | *Bittium reticulatum* (da Costa, 1778) |
| *Alvania* sp*.* (Risso, 1826) | *Cerithiopsis* sp*.* (Forbes & Hanley, 1850) |
| **Aplus dorbignyi* (Payraudeau, 1826) | Columbella rustica (Linnaeus, 1758) |
| *Bittium* spp. (Gray, 1847) | **Mangelia paciniana* (Calcara, 1839) |
| **Cerithium vulgatum* Pagured (Bruguière, 1792) | Triphora spp. (Blainville, 1828) |
| *Columbella rustica* (Linnaeus, 1758) |  |
| *Conus ventricosus* (Gmelin, 1791) | **Arthopoda Crustacea** |
| *Diodora gibberula* (Lamarck, 1822) | Amphipoda spp. (Latreille, 1816) |
| **Euthria cornea* (Linnaeus, 1758) | Isopoda spp. (Latreille, 1816) |
| **Gibberula miliaria* (Linnaeus, 1758) |  |
| *Nassarius semistriatus* (Brocchi, 1814) | **Annelida** |
| **Ocinebrina corallinoides* (Pallary, 1912) | **Lepidonotus* sp. (Leach, 1816) |
| **Pagurus* spp*.* (Fabricius, 1775) | **Polichaeta* spp*.* (Grube, 1850) |
| **Pusia tricolor* (Gmelin, 1791) |  |
| *Rissoa auriscalpium* (Linnaeus, 1758) |  |
| *Rissoa* spp*.* (Desmarest, 1814) |  |
| *Rissoella inflata* (Monterosato, 1880) |  |
| *Steromphala umbilicaris* (Linnaeus, 1758) |  |
| **Tarantinaea lignaria, Pagured* (Linnaeus, 1758) |  |
| Triphora spp. (Blainville, 1828) |  |
|  |  |
| **Mollusca Polyplacophora** |  |
| *Rhyssoplax olivacea* (Spengler, 1797) |  |
|  |  |
| **Arthopoda Crustacea** |  |
| Amphipoda spp. (Latreille, 1816) |  |
| Isopoda spp. (Latreille, 1816) |  |
| **Alpheus* sp*.* (Fabricius, 1798) |  |
|  |  |
| **Annelida** |  |
| **Lepidonotus* sp. (Leach, 1816) |  |
| **Polichaeta* spp*.* (Grube, 1850) |  |
