# Supplementary material for: Predation risk of the sea urchin Paracentrotus lividus juveniles in an overfished area reveal system stability mechanisms and restocking challenges
Source: PLoS One. 2024 Apr 18;19(4):e0301143. doi: 10.1371/journal.pone.0301143 (PMC11025834; doi:10.1371/journal.pone.0301143)
Supplement: Attachment — Submitted filename: SeaUrchinPredationReview.docx [file pone.0301143.s004.docx]

The study presented seeks to explore the relationships between juvenile *Paracentrodus lividus* recruitment and various predation pressures within different habitats, with the goal of improving the management of this heavily exploited taxon. This experimental study used tank raised juvenile *P. lividus* to comparatively assess survival rates in heavily vegetated (turf) and non-vegetated (barrens) natural habitats*,* where megapredators (length > 2 cm) were either present or excluded (via 40x40x40 cm cages w/ 1 cm mesh). Overall, the work performed by the authors appears to report significant results that should be of interest to a broad readership. The methodology is sound, and the interpretations and conclusions are well supported by the experimental data. There are some small issues regarding clarity and grammatical errors (see minor suggestions below), but the undisclosed restrictions regarding data availability are of main concern. Assuming that those issues (especially data availability) are adequately addressed, I support publication of the revised version of this manuscript in PLoS ONE.

Major Comments:

1. Data Availability
   a. Authors answered “No – some restrictions will apply” regarding data being fully available without restriction. It would pay well to elucidate the restrictions, and what this means regarding the ability to replicate the experiment and/or data analyses.

Minor comments: Clarity Suggestions

1. Introduction
   a. Lines 72–73 – “Accordingly, the recovery of the vegetated state would thus be impeded despite the removal of adult individuals, maintaining the barren state” – is unclear what the authors are trying to convey, perhaps a line along the lines of – “The lower predation rates of juvenile urchins would therefore hinder the recovery of the vegetated state and maintain the barrens, even in the absence of adult urchins”.
2. Materials and Methods
   a. Lines 135 – 137 – How were fertilized eggs and larvae counted during the incubation and raising of the juvenile urchins? Elucidating should only improve method clarity.
   b. Lines 151 – 152 – “they were transferred to outdoor tanks until they reached the size suitable for the release into the wild” – it is unclear as to what the designated “suitable” size is, a 15 mm size is mentioned for transition from indoor to outdoor tanks, but nothing about outdoor tanks to the field. Correction of this would improve ability to replicate the study.
   c. Lines 177 – 178 – “by counting in the field the juvenile sea urchin with daily visits during the first week and then with a weekly frequency.” – How were juvenile sea urchins in sites without predator exclusion cages kept within the study area? Correction of this would improve method clarity and the ability to replicate the study.
   d. Lines 182 – 183 – “and the predation rate (proxy of the predation risk) was expressed as 1-survival rate (expressed on a scale ranging from 0 to 1).” – how can we assume that predation is the only form of mortality in these juvenile urchins? – elucidation would improve method clarity.

Minor Comments: Grammatical/Typological Suggestions

1. Abstract
   a. Line 30 – “a key process structuring population” – suggested replacement – “a key population structuring process”.
   b. Line 31 – “despite sea urchin human harvest has quit” – suggested replacement – “despite an end to local human harvest of sea urchins”.
   c. Line 33 – “At this aim” – suggested replacement – “To facilitate this”.
   d. Line 37 – “survived recruits” – suggested replacement – “the recruits that survived”.
   e. Line 38 – “has” – suggested replacement – “had”.
   f. Line 40 – “survived recruits depending on the substrate” – suggested replacement – “the recruits that had survived, depending on the substrate”.
2. Introduction
   a. Line 76 – “countries,” – suggested replacement – “countries”.
   b. Line 82 – “last years” – suggested replacement – “over the last several years”.
3. Materials and Methods
   a. Line 177 – “by counting in the field the juvenile sea urchin” – suggested replacement – “by obtaining field counts of the juvenile sea urchins”.
